# Case report: Aborted sudden cardiac death as a first presentation of severe mitral annulus disjunction—a case series and review of the literature

**DOI:** 10.3389/fcvm.2023.1171226

**Published:** 2023-07-20

**Authors:** Fay Apostolou, Marios Ioannides, Andreas Mitsis, Constantina Koutsofti, Constantinos Deltas, Panayiotis Avraamides

**Affiliations:** ^1^ Department of Cardiology, Nicosia General Hospital, Strovolos, Cyprus; ^2^ Biobank.cy Center of Excellence in Biobanking and Biomedical Research, University of Cyprus, Nicosia, Cyprus; ^3^ School of Medicine, University of Cyprus, Nicosia, Cyprus

**Keywords:** mitral valve prolapse, mitral annulus disjunction, sudden cardiac death, ventricular arrhythmia, transthoracic echocardiography

## Abstract

Mitral annulus disjunction (MAD) is defined as a systolic displacement between the ventricular myocardium and the posterior mitral annulus supporting the posterior mitral leaflet. This structural abnormality is associated with the loss of mechanical annular function manifested as an abnormal systolic excursion of the leaflet hinge point into the left atrium but with maintained electrical function, separating the left atrium and ventricle electrophysiologically. The mitro-aortic fibrous continuity limits MAD anteriorly, between the aortic cusps and the anterior leaflet of the mitral valve. Consequently, MAD has been observed only at the insertion of the posterior leaflet. It can extend preferentially at the central posterior scallop. The first diagnostic modality aiding the diagnosis is transthoracic echocardiography (TTE), although in some cases adjunctive cardiac imaging modality might be suggested. MAD carries a strong association with malignant ventricular arrhythmogenesis and a profound predisposition for sudden cardiac death (SCD). In this context, a thorough investigation of this morphological and functional abnormality is vital in estimating the risk assessment and stratification for optimal management and elimination of the risk of the patient for SCD. Based on the current scientific data and literature, we will discuss the diagnosis, clinical implications, risk stratification, and therapeutic management of MAD.

## Introduction

Mitral valve prolapse (MVP) is the most frequent cause of primary or degenerative mitral regurgitation (MR) with estimated prevalence up to 3% in the general population (1, 2). Mitral annulus disjunction (MAD) is frequently coexistent with MVP, although the reported prevalence of MAD varies due to the different imaging modalities, various cut-offs, heterogeneous subpopulations, and various MR-severity grades (3–5).

The clinical significance of MAD in the absence of mitral valve disease is unknown, although it has been shown that the prevalence of ventricular arrhythmias in MAD with concomitant MVP and isolated MAD was not significantly different, which suggests the arrhythmogenicity of MAD alone (3, 6). MAD is currently considered an independent risk factor for ventricular arrhythmias and cardiac arrest independently of concomitant mitral valve abnormalities, suggesting the existence of a novel entity: MAD arrhythmic syndrome (7).

## Epidemiology

At first, MAD was described in autopsy studies to be present in approximately 6% of human hearts (4, 8). The prevalence of MAD among patients with MVP varies between 20% and 58% (3, 9, 10). In a certain subset of patients with myxomatous MVP, MAD prevalence varies between 21.8% and 98% (3–5). In patients with MAD, the reported presence of MVP was 78% (6).

In arrhythmic MVP syndrome, MAD prevalence is reported between 34% (11) in patients with Marfan syndrome and 40% in carriers of Loeys–Dietz syndrome (12). In the abovementioned collagen vascular diseases, MAD appears as a marker of higher severity of arrhythmic event frequency, a higher number for mitral valve intervention needed, and among patients with extensive MAD, even more arrhythmic events (11, 12). In isolated MVP with systematic MAD assessment, generally in younger patients, the prevalence of 30% of MAD was reported (first large prospective cohort) (9). The strongest MAD-associated MVP feature was the advanced myxomatous-degeneration (marked leaflet-redundancy and bileaflet-MVP). The association was independent of all listed baseline characteristics, whereas MR severity was not independently associated with MAD (9).

## Diagnostic criteria

The cornerstone for the diagnosis of MAD is cardiac imaging modalities. MAD can be detected easily in the long-axis views during end-systole using transthoracic echocardiography (TTE) (3–5, 10), and cardiac magnetic resonance (CMR) (13). As an adjunctive, cardiac computed tomography (CT) can also diagnose MAD. None of the abovementioned cardiac imaging modalities has been suggested in the present studies or guidelines as a gold standard method of diagnosis. In a study of 38 patients with myxomatous mitral valve disease, the authors described the recognition of MAD by using standard TTE using the length of the annular disjunction during end-systole on parasternal long-axis view, which was defined as the measurement from the junction of the left atrial wall and MV posterior leaflet to the basal LV posterior wall (4). With the use of a standardized intraoperative TEE protocol, MAD has also been described and defined as a separation between the P2 insertion into the left atrial wall and the atrioventricular attachment performed in a four-chamber mid-esophageal view. From this study, the significant correlation between the magnitude of disjunction and the number of segments with prolapse/flail was revealed (5), although according to a quantitative 3D echocardiographic study, more than one scallops of the posterior MVL may be involved (14).

MAD is widely known as a common abnormality in MVP. The disjunctive annulus is paradoxically functionally decoupled from the left ventricle and coupled more to the left atrium, consequently leading to paradoxical annulus dynamics with systolic expansion and annulus flattening. As a result, the abnormal mechanical stress and disjunctive annular dynamics exerted on the mitral valve and subvalvular apparatus accelerate the degenerative processes (14).

Excluding pseudo-MAD phenotype is of great importance. Pseudo-MAD refers to the juxtaposition of the posterior leaflet of the mitral valve on the atrial wall in systole mimicking MAD (15, 16). Particular attention is crucial for the recognition of MAD due to its strong correlation with life-threatening arrhythmic events. Therefore, high suspicion is suggested, especially in patients with concomitant MVP or myxomatous MV disease with arrhythmias or symptoms of arrhythmias. For the purpose of not underreporting such a potentially high arrhythmogenic entity, a multi-imaging modality approach may be necessary to raise the detection odds. Imaging modalities with superior spatial resolution such as cardiac CT or CMR may be used as adjunctive diagnostic tools for those with a lesser degree of MAD or high suspicion of MAD (17, 18). Cardiac CT can evaluate the mitral valve using a multiplanar reconstruction method, thereby providing detailed anatomic information, visualization of the entire circumference of the mitral valve attachment, and more sensitive detection of disjunction (17, 18). CMR is considered the gold standard imaging technique for evaluating myocardial function, quantifying chamber volumes, and detecting scar tissue/fibrosis (19) and may assist the risk stratification and prognostic information. Therefore, CMR may be an important adjunct to echocardiography as it can better define more subtle MAD and detect the markers of arrhythmia risk (13, 20).

Due to the high prevalence of MAD in normal subjects, an acceptable cut-off value of separation (≥5 mm) between the posterior leaflet insertion and the atrioventricular junction (basal LV free wall) is approved based on a histologic (7) and an echocardiographic description (5).

## Arrhythmia substrate

The proposed mechanism of arrhythmias in MAD is related to a combination of papillary muscle fibrosis- anatomical substrate and mechanical stretch of the myocardium, which has been supported by CMR imaging findings (6, 21, 22).

## SCD prevalence

All patients with MVP should be mandatory stratified for sudden cardiac death (SCD) risk. Risk stratification of patients with MVP includes focused history, 12-lead ECG, extended ECG monitoring, and detailed echocardiography. The use of CMR and implantation of a loop recorder are more selective depending on the probability of VAs. Risk stratification involves two phases based on the clinical and imaging context and the detected arrhythmia (23). Even though the risk of malignant arrhythmias and SCD in MVP patients is low, estimated approximately 0.2%–0.4% per year, the real prevalence seems to be underestimated (24). A comprehensive clinical evaluation is mandatory to identify MVP patients with higher risk of arrhythmias. Family history of SCD is relevant to suggest the possibility of inherited arrhythmia syndromes (long-QT syndrome, arrhythmogenic right ventricular cardiomyopathy) as alternative diagnoses. Family history of MVP should also raise the attention to heritability, as either sporadic or familial forms.

Several phenotypic risk features of MVP have been identified including young age, female sex, high burden of ventricular ectopy (VE), bileaflet myxomatous MV degeneration, MR severity, and flail leaflet (8, 25).

More malignant risk factors of arrhythmia in MAD are younger age, high burden of VE, longer longitudinal distance of MAD, and evidence of papillary muscle fibrosis-anatomical substrate on CMR (6, 13). In addition to the arrhythmia risk, the presence of MAD has been shown to be associated with greater extent of mitral leaflet and chordae tendineae deformity and LV enlargement, and MR severity correlates with the degree of MAD. In other words, MAD may be a contributor to the progression of MR and may predispose to future degeneration and prolapse in patients without concomitant MV disease (9, 14). It still remains unclear whether MAD depth is a predictor of more frequent arrhythmic events, as it is proposed by the MAD-associated arrhythmia mechanism and the myocardial fibrosis as a hypothetic causative. Even though MAD presence in subjects with MVP has raised the VAs odds during follow-up, the first 10 years of follow-up did not confirm an association with excess mortality. As it is suggested by the risk stratification strategy in patients with MVP, the aim is to assess the risk of VAs and SCD according to the phenotypic risk features [T wave inversion in inferior leads, multiple polymorphic premature ventricular captures, MAD, redundant MV leaflets, enlarged left atrium, left ventricular ejection fraction (LVEF) < 50%, late gadolinium enlargement (LGE)] and the presence of high-risk ventricular tachycardia (VT). Based on the clinical and imaging context and the uncovered arrhythmia, Holter monitoring (periodic/diagnostic) and implantable loop recorder (ILR) are used for the risk stratification (23). MAD requires a deep and thorough understanding due to its apprehensive arrhythmogenic trait, and even the diagnosis of isolated MAD solely should instead raise awareness for a careful follow-up and not to acknowledge this as a high-risk factor for sudden death in all patients.

## Case report 1

**Figure 1:**
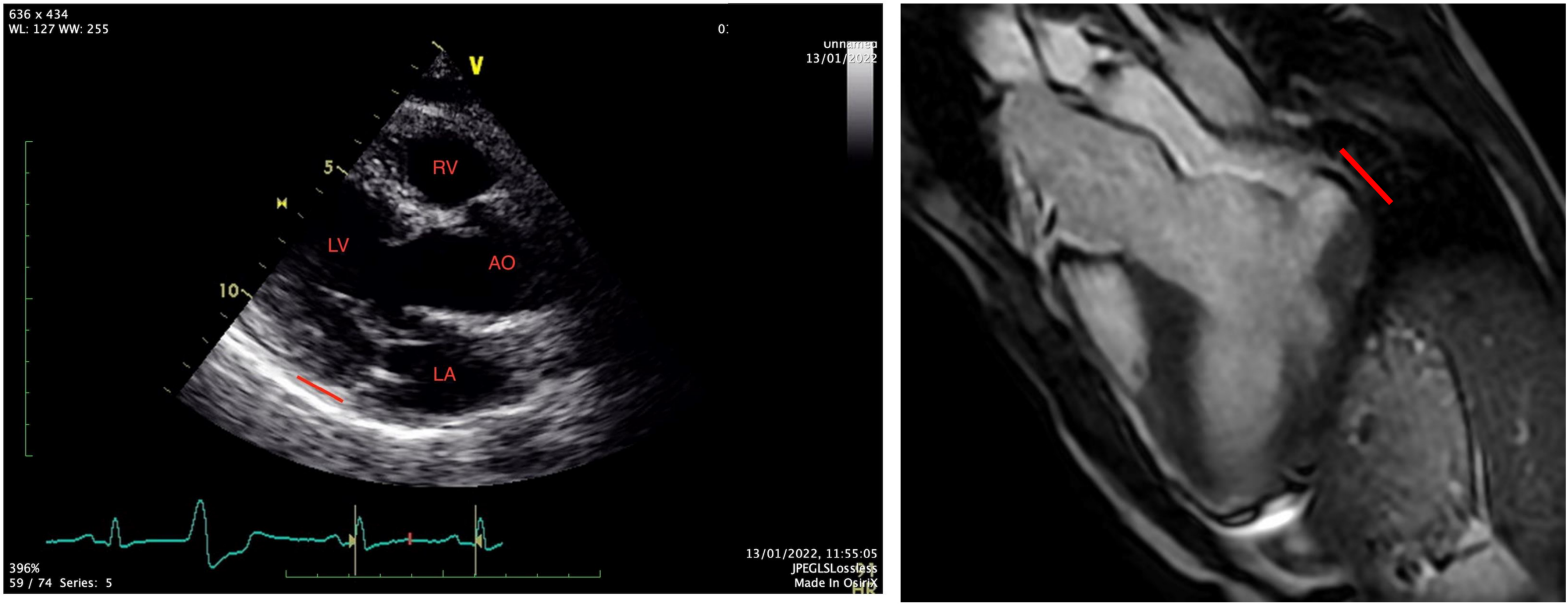
TTE and MRI, MAD noted with the red line.

A 31-year-old Caucasian with a known medical history of bronchial asthma, mild MVP, autonomic dysregulation of sinus and atrioventricular node, smoking, and a family history with MVP and SCD was transferred to the emergency department of our hospital, following an episode of out-of-hospital cardiac arrest due to ventricular fibrillation. After the restoration of spontaneous circulation, a thorough clinical examination showed unremarkable findings, and a bedside FOCUS TTE on presentation showed a severely depressed LVEF and coronary angiography excluded obstructive coronary artery disease (CAD). Repeated TTE and TOE showed improved LV function (50% EF) and evidence of bileaflet MVP, severe mitral regurgitation, and MAD approximately 16 mm, not clearly visualized on initial imaging (Figure 1). The CMR showed significant annulus disjunction of the posterior leaflet of MV (Figure 1), measuring up to 16 mm, severe bileaflet MVP, and severe MR with posteriorly directed eccentric jet (Supplementary Video S1). Additional finding was an extensive, disseminated fibrosis of the mid lateral and inferolateral LV wall, posteromedial papillary muscle revealed by late gadolinium enhancement.

A genetic test was performed with a next generation sequencing panel and revealed a very rare genetic variant in the *MYOM1* gene, classified as a variant of unknown significance (VUS). No specific relation between this mutation and MAD has been described.

The patient was discharged after receiving a secondary prevention implantable cardioverter defibrillator (ICD) and was referred for mitral valve repair. The post-operation follow-up with a cardiac event monitor showed a subtle arrhythmogenic activity VE burden (approximately 1.0%) and mild mitral regurgitation on transthoracic echocardiogram. He remained stable and asymptomatic.

## Case report 2

A 50-year-old Caucasian man has been transferred to our clinic from another hospital, in an emergency manner for emergency coronary angiography due to persistent monomorphic ventricular tachycardia. While evaluating the patient, the patient collapsed abruptly due to ventricular fibrillation. He was successfully resuscitated from cardiac arrest and transferred fully consciousness for emergency coronary angiography. The clinical evaluation prior to the coronary angiography showed no significant clinical findings.

His past medical history was smoking in a habitual manner and former drug user (drug test upon admission was negative), reporting abstinence for 3 years now. He mentioned occasional palpitations without any further investigation. Collecting data from his family history, one case of sudden death was reported in the family at the age of 60 in a second-degree relative. Family history was negative for primary electrical diseases and MVP. He was not taking any medications or drugs at the time of presentation.

The coronary angiography excluded obstructive coronary artery disease as the cause of cardiac arrest and revealed a moderately impaired LV systolic function by left ventriculography.

**Figure 2:**
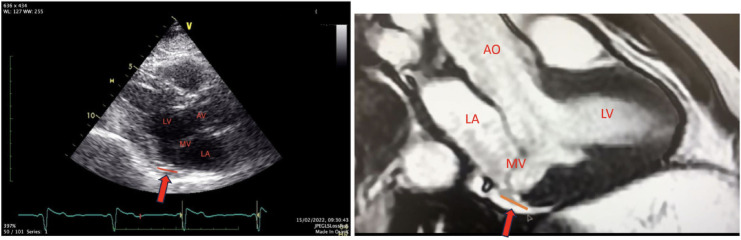
Transthoracic and MRI long-axis views. The red line and red arrow indicate the MAD area.

**Figure 3:**
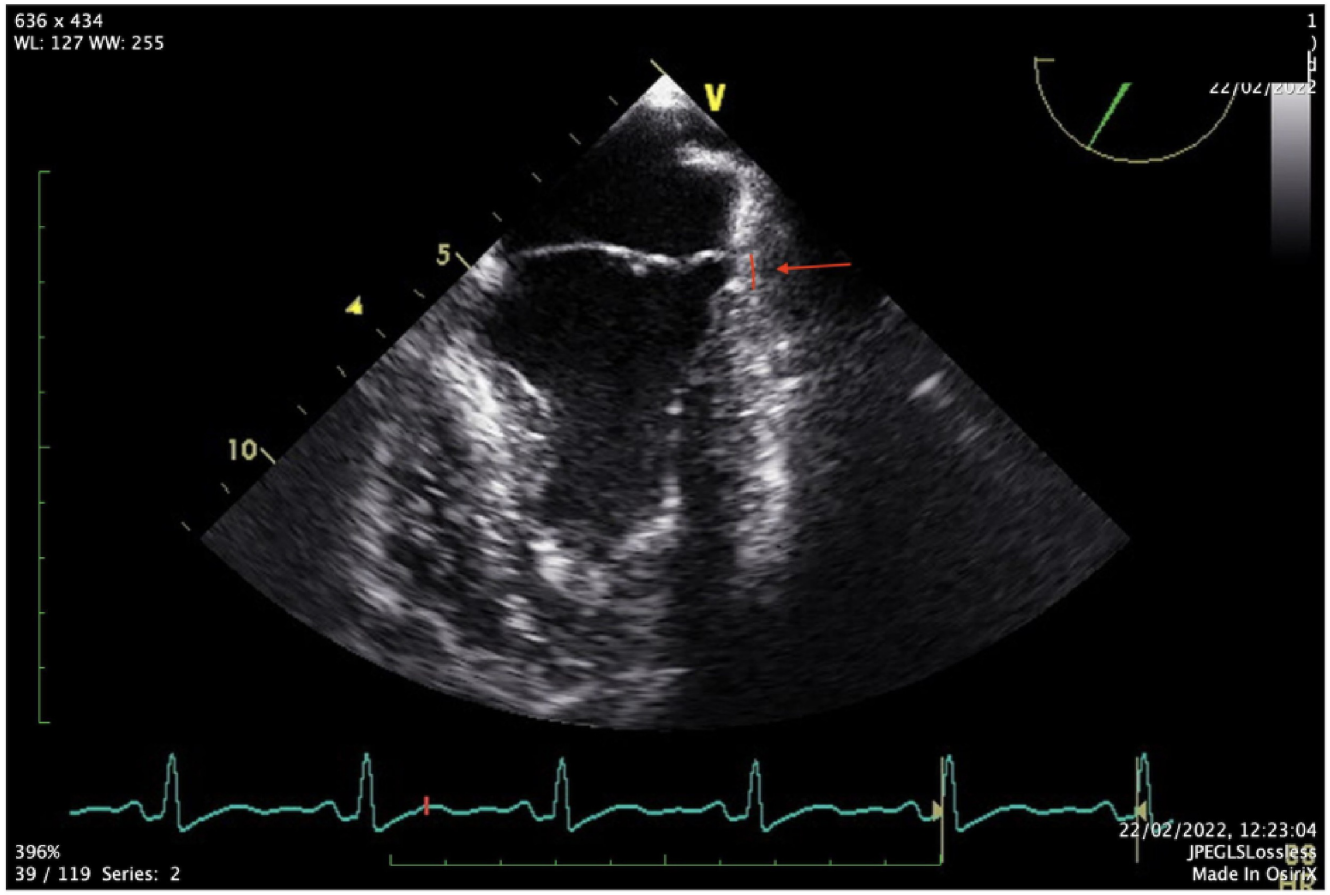
Transesophageal view of MAD (red line and red arrow).

The FOCUS echocardiographic assessment showed a moderately impaired LV systolic function, gross estimation LVEF: 40%, without pericardial effusion, and intact proximal ascending aorta. A complete TTE and TEE study that followed revealed a mildly dilated LV with borderline normal LVEF and MAD (Figures 2,3, Supplementary Videos S2, S3).

The cardiac magnetic resonance report an unveiled annular disjunction of the posterior LV wall, measuring a longitudinal MAD distance of 8 mm (Figure 2), with late gadolinium enhancement due to fibrosis in the anterolateral papillary muscle and basal posterolateral left ventricle. The left ventricular ejection fraction was borderline normal (LVEF: 50%), with mildly dilated left ventricular dimensions (LVESD: 36 mm/LVEDD: 53 mm short axis).

A multidisciplinary heart team suggested an ICD implantation as a secondary prevention for SCD and no further surgical intervention due to limited data in the current literature.

A genetic investigation with next generation sequencing panel revealed two variants of unknown significance (VUS) in *ANK2* and *TTN.* Both variants have no specific relation with MAD.

The patient is currently asymptomatic and without any worrying arrhythmic findings on follow-up cardiac rhythm monitoring 2 months after. His ICD check is also without any remarkable arrhythmic activity.

## Risk stratification

Beyond the first part of the diagnostic algorithm with clinical approach, imaging, electrocardiographic, and cardiac event monitoring, asymptomatic patients with MAD—with or without MVP—require a close follow-up with repeated 2D-transthoracic echocardiography over the years, to explore MAD development over time and for routine rhythm surveillance, as MAD is an independent risk factor for arrhythmias (21). Additional diagnostic modalities such as CMR and electrophysiological study might be necessary for further risk stratification in cases with known risk factors for malignant arrhythmia. CMR is an optimal method for evaluating cardiac and valvular structure and function and for assessing the presence and extent of myocardial fibrosis. The presence of LGE is most often observed in the papillary muscles or basal inferior wall and is highly linked with an increased risk of arrhythmias (26). The early detection of high-risk features is crucial in risk stratifying the patients and determining the necessity for more extensive evaluation and appropriate therapeutic management.

## Therapeutic management

Current literature regarding the medical management of AMVP patients is scanty. The medical management should be addressed to the specific presentation by aiming the symptomatic improvement and improving survival (23).

The American Heart Association/European Society of Cardiology guidelines for ventricular arrhythmias and SCD have no distinctive criteria or recommendations for the secondary prevention of SCD and the management of ventricular arrhythmias in significant mitral valvulopathy (27, 28).

The pivotal challenge of SCD prevention mandates the establishment of primary and secondary preventive methods such as screening programs, technological measures (ECG, echocardiogram), and a fundamental risk stratification strategy with the use of CMR, treadmill exercise test, and a serial rhythm monitoring. In the case where MVP had been diagnosed in a random order or screening (relatives of SCD victims or athletes), a risk stratification strategy may be essential depending on the existence of high-risk criteria. It is essential to thoroughly evaluate with electrophysiological study, CMR, and echocardiography, particularly the young individuals, for the proper selection of candidates for ICD implantation (27).

According to the latest data from the expert consensus, patients with AMVP and a documented history of VF or hemodynamically not tolerated VT, in the absence of reversible causes, should receive an ICD. MVP with LVEF <35% and symptomatic HF despite ≥3 months of OMT should receive an ICD.

Ablation of PVCs in patients with frequent PVCs who are symptomatic or have decreased LV function is advised. Ablation of VA in MVP patients should be performed in experienced centers with expertise in VA ablation and interventional and surgical treatment of MV regurgitation. Ablation of PVCs is reasonable if triggering VF, particularly if not controlled by medications. Ablation of sustained monomorphic VT despite antiarrhythmic treatment or if antiarrhythmic treatment is not desired, or contraindicated, should be performed in MVP patients with recurrent ICD therapies. Mitral valve surgery may reduce the burden of malignant VAs in MVP patients and severe MR (23).

The promising potential of biomarkers and genetic studies for the determination of high-risk individuals, particularly after the latest correlating with familial clustering, is likely to be further tested in the coming future (29, 30).

A case report emphasized and illustrated the need to maintain alertness in malignant MVP patients and signified the role of mitral valve repair in the treatment of ventricular arrhythmias resulting from malignant MVP as a potential treatment option and secondary preventive method for SCD (31). Currently, there is no preferable surgical approach (valve repair or replacement), and as with the available data, none of them appears superior in terms of the reduction of ventricular arrhythmia burden. Despite this, a complete resolution of the disjunction can be achieved in both procedures and, subsequently, the reduction of malignant arrhythmias (13).

## Conclusion

MAD is an anatomical, congenital variation associated frequently with MVP. Distinct anatomic characteristics of this potentially lethal entity may increase the risk of malignant ventricular arrhythmia and SCD. A suggestive therapeutic approach with ICD implantation and mitral valve repair might be necessary for this appropriate group. Large cohorts with long-term follow-up are required to better define MAD without MVP and its outcome and consequences. Future prospective studies evaluating ECG features, ventricular arrhythmia burden and morphology, and electrophysiological findings may provide further information. In individuals with different MAD phenotypes, further evidence could clarify its arrhythmogenic role and specify the effectiveness of antiarrhythmic therapy, the potential use of early catheter ablation in refractory ventricular arrhythmias, and the surgical approach in managing ventricular arrhythmia in patients with MVP (32).

## Data Availability

The raw data supporting the conclusions of this article will be made available by the authors, without undue reservation.
